# Aneurysm of Portosystemic Fistula: A Case Report and Review of Literature

**DOI:** 10.5005/jp-journals-10018-1243

**Published:** 2017-09-29

**Authors:** Sila Ulus, Gulhan Ertan Akan, Cengiz Erol

**Affiliations:** 1 Department of Radiology, Acibadem University School of Medicine, Istanbul, Turkey; 2 Department of Radiology, Istanbul Medipol University School of Medicine, Istanbul, Turkey

**Keywords:** Aneurysm, Computed tomography angiography, Spontaneous portosystemic venous fistula, Ultrasound.

## Abstract

**Aim:**

A case of asymptomatic aneurysm of spontaneous portosystemic venous fistula (SPVF) with the radiologic findings is described.

**Background:**

Although advances and more widespread use of ultrasound (US) and computed tomog -raphy angiography (CTA) have enabled more detection of SPVF in the liver, it is a rare entity.

**Case report:**

A 49-year-old male was referred to our hospital’s nephrology outpatient clinic due to hypertension. Abdominal sonography examination detected a well-defined cystic lesion adjacent to the middle hepatic vein in the liver. The lesion showed venous flow in the color Doppler US examination. Computed tomography angiography examination revealed an aneurysm of the fistula.

**Conclusion:**

Radiologists should be aware of this vascular anomaly and cyst-like lesions in the liver should be examined with color Doppler ultrasonography for possible vascularization, and be differentiated with CTA if necessary.

**Clinical significance:**

This condition is usually encountered incidentally and patients usually have no symptoms. However, severe complications, such as hepatopulmonary syndrome, liver tumors, encephalopathy, and heart failure can be seen.

**How to cite this article:** Ulus S, Akan GE, Erol C. Aneurysm of Portosystemic Fistula: A Case Report and Review of Literature. Euroasian J Hepato-Gastroenterol 2017;7(2):178-180.

## INTRODUCTION

Although advances and more widespread use of US and CTA have enabled more detection of SPVF in the liver, it is a rare entity.^1^ These have been categorized into four different morphologic types by Park et al.^2^ Type I includes patent paraumbilical veins, located in the liver. Type II is a localized peripheral shunt in which single or multiple communications are found between peripheral branches of portal vein and hepatic veins in one hepatic segment. Type III is aneurysmal, with peripheral portal and hepatic veins being connected through an aneurysm. Type IV has multiple communications between peripheral portal and hepatic veins diffusely in both lobes.

The cause of SPVF is unknown and controversial. In the absence of cirrhosis and portal hypertension, a congenital origin is possible. Here we present a rare case of asymptomatic aneurysm of the SPVF with the radiologic findings.

## CASE REPORT

**Figs 1A and B:**
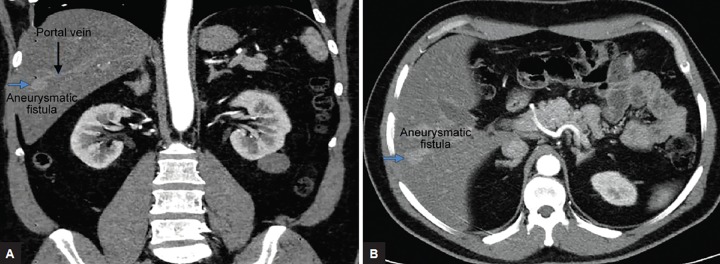
Coronal (A) and axial (B) CTA images reveal an aneurysmatic fistula (blue arrow) approximately 2 cm in size in the intersection of segment 5 to 6 of the right lobe, between the right portal vein (black arrow) and the right hepatic vein branches in the liver

A 49-year-old male was referred to our hospital’s nephrology outpatient clinic due to hypertension. Physical examination showed no anomaly, and the abdomen was soft. Laboratory data showed normal blood counts, liver and renal function tests, glucose, and lipid levels. Abdominal sonography (US) examination was requested to evaluate the kidneys and it revealed an incidental, 1.5 × 1.5 cm in size, well-defined anechoic lesion adjacent to the middle hepatic vein in the liver. The lesion showed spectral imaging features consistent with venous flow in the color Doppler US examination. Parenchymal echogenicity of the rest of the liver was normal and there was no focal parenchymal lesion. The main portal vein was patent and the course of the right and left portal veins was normal. The liver and spleen were normal in size. The patient had no history of abdominal surgery or trauma or liver biopsies. Computed tomography angiography examination revealed an aneurysmatic fistula approximately 2 cm in size in the intersection of segments 5 and 6 of the right lobe, between the right portal vein and the right hepatic vein branches in the liver (Fig. 1). No intervention was performed because the patient did not have any symptoms. The patient is in follow-up to rule out any increase in the size of the SPVF.

## DISCUSSION

Intrahepatic portosystemic venous fistula is defined as a connection between the intrahepatic portal vein and systemic vein due to an anomaly of the intrahepatic venous channels. Intrahepatic fistula between the portal and systemic vein can be congenital or secondary to cirrhosis, trauma, or portal vein aneurysm rupture. The most common type is seen between the portal vein and extrahepatic systemic veins (perihepatic veins or inferior vena cava) in patients with portal hypertension due to cirrhosis. Fistula between the portal vein and hepatic vein is less frequent than the fistula between the portal vein and perihepatic veins or inferior vena cava. These fistulae are usually smaller than 2 cm. Big fistulas are usually congenital. A fistula between the portal vein and the hepatic vein is assumed to be spontaneous or congenital without the coexistence of liver disease or trauma.^3^ In the literature, these fistulas are speculated to occur from persistent embryonic venous anastomoses due to inadequate regression of the connections between the vitelline veins.^4-6^ Another theory is the rupture of the portal vein aneurysm into the hepatic vein.^6^

This condition is usually encountered incidentally and patients are usually symptom free. However, severe complications, such as hepatopulmonary syndrome, liver tumors, encephalopathy, and heart failure can be seen. In symptomatic cases where hepatic encephalopathy is seen in the cirrhotic background, treatment modalities, such as transcatheter embolization, shunt ligation, or hepatic resection can be used.^7-10^

Color Doppler imaging is the first imaging modality to investigate for this entity. It reveals the blood flow between the portal vein and the hepatic vein in the presence of intrahepatic portosystemic venous shunts. Furthermore, spectral imaging of the lesion can demonstrate the continuous waveform in the portal vein, turbulence in the aneurysmal cavity, and the biphasic waveform in the hepatic vein or extrahepatic systemic vein.^5^ The diagnosis of intrahepatic portosystemic venous shunts can also be made by CTA, magnetic resonance imaging, or conventional angiography.

The vascular malformation we present is presumably a congenital anomaly, since no signs of cirrhosis or trauma were identified. The CTA was performed to confirm the diagnosis. According to the classification by Park et al,^2^ our patient had an aneurysmatic type III connection between the right portal vein and the right hepatic vein branches. No treatment was performed since the patient did not have any symptoms or findings, and he was under follow-up.

## CONCLUSION

In conclusion, radiologists should be aware of this vascular anomaly, should evaluate the cyst-like anechoic lesions in the liver with color Doppler ultrasonography for possible vascularization, and when needed differentiate these entities with CTA.

## CLINICAL SIGNIFICANCE

This condition is usually detected incidentally and patients usually have no symptoms. However, severe complications, such as hepatopulmonary syndrome, liver tumors, encephalopathy, and heart failure can be seen. Advanced imaging modalities like CTA can help in the differentiation of patients who need treatment from asymptomatic patients with no underlying liver diseases.
